# Balanced difficulty task finder: an adaptive recommendation method for learning tasks based on the concept of state of flow

**DOI:** 10.1007/s11571-020-09624-3

**Published:** 2020-08-27

**Authors:** Anis Yazidi, Asieh Abolpour Mofrad, Morten Goodwin, Hugo Lewi Hammer, Erik Arntzen

**Affiliations:** 1 Department of Computer Science, Oslo Metropolitan University, Oslo, Norway; 2 Department of Computer Science, University of Agder, Kristiansand, Norway; 3 Department of Behavioral Science, Oslo Metropolitan University, Oslo, Norway; 4 Simula Metropolitan Center, Oslo, Norway

**Keywords:** Adaptive task difficulty, State of flow, Intelligent tutoring system, Game ranking systems, Online learning, Adjusting delayed matching-to-sample, Computerized adaptive testing, Stochastic point location

## Abstract

An adaptive task difficulty assignment method which we reckon as balanced difficulty task finder (BDTF) is proposed in this paper. The aim is to recommend tasks to a learner using a trade-off between skills of the learner and difficulty of the tasks such that the learner experiences a state of *flow* during the learning. Flow is a mental state that psychologists refer to when someone is completely immersed in an activity. Flow state is a multidisciplinary field of research and has been studied not only in psychology, but also neuroscience, education, sport, and games. The idea behind this paper is to try to achieve a flow state in a similar way as Elo’s chess skill rating (Glickman in Am Chess J 3:59–102) and TrueSkill (Herbrich et al. in Advances in neural information processing systems, 2006) for matching game players, where “matched players” should possess similar capabilities and skills in order to maintain the level of motivation and involvement in the game. The BDTF draws analogy between choosing an appropriate opponent or appropriate game level and automatically choosing an appropriate difficulty level of a learning task. This method, as an intelligent tutoring system, could be used in a wide range of applications from online learning environments and e-learning, to learning and remembering techniques in traditional methods such as adjusting delayed matching to sample and spaced retrieval training that can be used for people with memory problems such as people with dementia.

## Introduction

Attempts to achieve computer tutoring systems that are as effective as human tutors can be traced back to the earliest days of computers (Smith and Sherwood 1976). *Online learning* is becoming a significant driving force in today’s educational systems. The lack of faculty members is a common trend in today’s universities which makes personalized *one to one* teaching challenging, or practically impossible. Students may struggle to fulfill their full potential because the assigned tasks are generic and not tailored to their specific needs and skill level. Several studies show that personalized learning is the key to increased fulfillment of potential (see, e.g., Miliband 2004). A possible solution to the latter problem is resorting to the advances in *AI* in order to personalize the teaching process. AI could be defined as: “*The automation of activities that we associate with human thinking, activities such as decision-making, problem solving and learning”* (Bellman 1978).

Some of early studies that allude to the term *Intelligent Tutoring System (ITS)* dates back to 1982, where D. Sleeman and J.S Brown pioneered the idea of a system designed to help students reach their full potential in a limited amount of time (see Sleeman and Brown 1982). A few years later, a study is published demonstrating that individual tutoring is twice as effective as group teaching (Bloom 1984). Later, online e-learning platforms such as *Kahn Academy*^1^ and *Udemy*,^2^ massive open online course (MOOC) such as *MIT OpenCourseWare*,^3^ digital hand in tools like *Fronter*, plagiarism controls like *Ephorus (Fronter)*, and autograding assignment tools such as *Bakpax*^4^ have emerged. True ITS also exists with open tools like *Codeacademy*^5^ and other e-learning platforms.

ITSs can raise student performance beyond the level of traditional classes and even beyond the level of students who learn from human tutors (see Kulik and Fletcher 2016, for a survey). A recent study by Chirikov et al. (2020) shows that online education platforms could scale high-quality science, technology, engineering, and mathematics (STEM) education through national online education platforms at universities. Such instruction can produce similar learning outcomes for students as traditional, in-person classes with a much lower cost (see also VanLehn 2011, for a review of relative effectiveness of human tutoring, intelligent tutoring systems, and other tutoring systems or no tutoring).

An ITS is supposed to *“provide immediate and customized instruction or feedback to learners”* (Psotka et al. 1988). In this paper, we provide algorithms that aspire to fulfill the latter statement for the purpose of task selection.

Many ITSs are based on *Computerized Adaptive Testing (CAT)* which is a form of computer-based test in which the correctness of the student’s responses shapes the difficulty level of upcoming tasks (see, e.g. Hatzilygeroudis et al. 2006; Kozierkiewicz-Hetmańska and Nguyen 2010; Jansen et al. 2016, for instance). The aims of testing and practicing through tutoring differ; testing should efficiently estimate the student’s ability (Birnbaum 1968; Eggen and Verschoor 2006), while training and practicing need to consider motivation and involvement of students in line with the length of the test (Jansen et al. 2016). A probability of success of 0.5 could minimize the test length, but this level of challenge could be frustrating for some students. For instance, in *Math Garden*, which is a web-based application for monitoring and practicing math skills based on CAT principles (Klinkenberg et al. 2011), a success rate of 75% is considered on average.

There is a substantial body of work on *Learning Automata (LA)* and ITSs (see, e.g. Oommen and Hashem 2013). In simple terms, LA is a stochastic machine attempting to find the optimal strategy from a set of actions in a random environment. LA, as a fundamental problem in AI, is particularly important in decision making under uncertainty (see Narendra and Thathachar 2012, for an introduction to LA). The term tutorial-like systems refers to study tutorial systems while no entity needs to be a real-life individual. Research in this field tries to model components of the system with appropriate learning models, such as LA (Oommen and Hashem 2013).

In a tutorial-like system, the teacher also might be stochastic and learns through the process of training (Hashem 2007). The design and analysis of a tutorial-like system model could involve modeling of a student (Oommen and Hashem 2009b), modeling of a classroom of students where artificial students can interact and learn from each other as well as the teacher (Oommen and Hashem 2009a), modeling of a (stochastic) teacher (Hashem and Oommen 2007), modeling the domain knowledge (Oommen and Hashem 2010), and modeling how teaching abilities of a teacher can be improved (Oommen and Hashem 2013).

ITSs can also be applied in some traditional learning methods in behavior analysis such as titrated delayed Matching-to-Sample (MTS) method, also referred as adjusting delayed MTS (Cumming and Berryman 1965; Sidman 2013).^6^ Titrated delayed MTS has been used to study remembering in a variety of settings, including to study important variables in analyzing short-term memory problems (Arntzen and Steingrimsdottir 2014). Similar applications of ITSs in MTS and titrated delayed MTS procedures, can proposed to the computational models of these experimental methods which are usually introduced in the sake of research (see, e.g. Mofrad et al. 2020, for a recent computational model that simulates MTS procedure). ITSs can be used as a tool in the simulation part of training phase of MTS or titrated delayed MTS procedures to study the effect of adaptive training in a simulator model.

Spaced retrieval training (SRT) (Camp et al. 1989) is another method of learning and retaining a piece of information by recalling that piece of information over increasingly longer intervals. The underlying problem in SRT is also similar to the adaptive difficulty task assignment which is addressed here. The SRT method is especially used for people with dementia (Camp et al. 1996).

Note that defining or measuring *task difficulty* can be addressed in many ways. A definition based on whether or not a task is performed, has applications in developmental research. In this context, easier tasks can be performed at earlier stages of development (see, e.g. Gilbert et al. 2012). For healthy adults, a difficult task can be defined as a quantitative measure, say percentage of task compliance in a series of trials. Response time is another measure of task difficulty, where a longer response time in average is equivalent to a more difficult task. Accuracy and response time, however, trade against each other (Fitts 1966; Wickelgren 1977) and both must be considered in a well-defined and standard task difficulty measure. Difficult tasks in this respect, can be defined as those with long response time and and/or high frequency error (see, e.g. Gilbert et al. 2012, for other accounts in defining task difficulty).

In this paper, we present a formal theory by which an ITS can select the difficulty of task in a similar manner to selecting an opponent of similar capabilities in *balanced difficulty game* (Herbrich et al. 2006), which is called Balanced Difficulty Task Finder (BDTF). As suggested by systems such as Elo’s chess skill rating (Glickman 1995) and TrueSkill (Herbrich et al. 2006) for matching game players, matched players should have similar capabilities and skills in order to achieve a balance between skills and challenges to experience the state of flow. We draw analogy between choosing an appropriate opponent or appropriate game level and automatically choosing an appropriate level of a learning task. It is noteworthy that by way of analogy, we can model the student as the player and the chosen task by the system as the opponent.

### Paper organization

The remainder of this paper is organized as follows. “State of art” section reviews the state of the art and various approaches to ITS modeling. “Modeling task selection as balanced game using balanced difficulty task finder” section models task selection as balanced difficulty game by resorting to our devised BDTF. “The concept of flow” section addresses the concept of flow from psychological point of view. In “Related work on games” section, related works from research on games are reported. “Neural basis of adaptive learning and state of flow experience” section addresses some literature on neural basis of adaptive learning and state of flow. Furthermore, theoretical formulation of BDTF is provided in “Formulating learning as a balanced difficulty game” section. Experimental results in “Experimental results” section catalogues the convergence properties of the BDTF discussed in the theory part. Finally, concluding remarks and future works are addressed in “Conclusions and future work” section.

## State of art

In this section, relevant studies and papers are discussed to give the reader an overview over the current state of the art. Although several papers on this topic exist dating back several years, the literature reviewed in this section is limited to content published (preferably) after 2005.

There are several approaches to create an ITS. In the most recent papers, we are presented with a mix of different artificial intelligence approaches to solve the problem. Common for most of the papers reviewed is the need for a model of student including different properties like learning-rate, previous experience and knowledge, and other variables. An approach for such a model (from now referred to as the *student model*) is represented in numerous studies (see for instance Brusilovsky and Millán 2007; Clement et al. 2014, 2015; Millán et al. 2010).

The use of the student model in recent papers suggests that this approach is fairly common in the field of ITS. Even though the model itself is fairly common, the implementation varies significantly. As an example, Clement et al. (2015) resort to a combination of a student model and a cognitive model to create a tutoring model. With this approach, the authors try to eliminate the need for a strongly typed student model. The goal is to adjust the learning tasks to individual students with as little information as possible. The use of a Learning Automata (LA) algorithm enables the system to find the optimal learning sequence for a specific student subject to some constraints; such as requiring certain activities to happen before others. A disadvantage of the latter approach is particularly the assumption that some tasks should be carried out in an order. The authors (Clement et al. 2015) assume that after task A1, either A2 or B1 need to follow. If students move to B1, they can not move back to any task in A category. This is in most cases a simplification of the learning process, since students should be able to work on several categories and practice by repeating previous categories.

Clement et al. (2015) use *partial-observable Markov decision process (POMDP)* for optimization of task selection, which is inspired by Rafferty et al. (2011) who used the students acquisition level to propose activities. This method requires the system to assume all students learn in the same way. It is also stated that this approach can be optimal, but requires sophisticated student and cognitive models. In most cases these methods are based on *knowledge tracing-methods (KTM)* which attempt to estimate student knowledge in a parametric manner. Usually, the lack of data causes this form of modeling to be inaccurate. POMDPs also has been suggested to be used for modeling a population of students, instead of individuals. This approach has been proven to be suboptimal in an ITS setting (Clement et al. 2015; Lee and Brunskill 2012).

On the other hand, several improved versions of the KTM have been proposed in the literature. A Representative example is the *Bayesian knowledge tracing* *(BKT)* with skill-specific parameters for each student. There are strong indicators that BKT models accounting for the student variance is superior to the Bayesian knowledge model (Pardos and Heffernan 2010; Yudelson et al. 2013). This partially nuances the criticism proposed by Clement et al. (2015).

A significant number of studies indicate that intrinsically motivated students perform better. Thus, this requires a good ITS keeps motivating the student throughout the whole learning experience. Lumsden (1994) investigated the optimal strategy for motivating the student, and found that one of the main keystones for a motivational experience is task mastery. This is backed up by Clement et al. (2015) who proposes a solution where the student is presented with tasks that are neither too easy nor too hard, but slightly beyond their current abilities. Psychologists refer to this experience as state of flow (see, e.g. Csikzentmihalyi 1996).

In this article, we propose a solution where each student starts with a predefined *optimal-difficulty* (Clement et al. 2015) which will be adjusted over time based on the student answers. Some students may be more prone to be motivated with challenging tasks, and therefore the overall learning outcome may be more effective for these students. On the other hand, we might find students struggling with the default or optimal-difficulty. In such cases, the learning-rate should be decreased, allowing these students to participate at a slower pace.

There are several possible alternatives to design an ITS. We have looked at several candidates in this study, including *multi-armed bandits* (Clement et al. 2015), *Bayesian-networks* (Millán et al. 2010) and *neural-networks* (Zatarain Cabada et al. 2015), each with its own advantages. As mentioned earlier the student model is an important part of this ITS. In the latter reviewed papers, the neural network and Bayesian-network both relied on comprehensive student models, with a solid core of data in order to be able to draw accurate assumptions and decisions. These systems are shown to be reliable and effective, but comprehensive data models are required in order to achieve optimal operation (Clement et al. 2015). With the use of LA it is possible to eliminate the need for prior-knowledge about the students. The LA is efficient, and it requires a weaker link between student and the cognitive model. Clement et al. (2015) propose an LA for seven to eight years old school-children learning to decompose numbers while manipulating money. Even though a generic solution is presented by Clement et al. (2015) relying on multi-armed bandit, there is no evidence that a similar approach is viable for use for adults and contexts addressed in online learning (see also Hashem and Oommen 2007; Hashem 2007; Oommen and Hashem 2009a, b, 2010, 2013, for LA based models for a generalized framework of tutoring system, called tutoring-like systems).

A limited number of studies describe the use of ITS in programming courses. As representative studies, we identified *Java Sensei* (Zatarain Cabada et al. 2015) and *ASK-ELLE* (Jeuring et al. 2012), each of the latter studies use a different machine learning approach. Java Sensei resorts to a combination of neural-network strategies and emotion sensors to register information and to make decisions based on input. ASK-ELLE ITS utilizes a domain reasoner using a Haskel Compiler called *Helios*. This compiler was developed to give feedback on wrong syntax. The system requires each student to complete a given task, but helps the student to accomplish the tasks by giving hints and examples relevant to found error(s).

Before moving to the model and contribution of this paper, we refer to the Stochastic Point Location (SPL) problem which has some similarities to the current work. A considerable amount of literature has been published on SPL since the Oommen work (Oommen 1997) (see for instance Yazidi et al. 2014; Mofrad et al. 2019). In SPL, an LA search for a point location in a line through the guidance of an external environment which might give faulty advice. Many scientific and real-life problems can be modeled as the instances of SPL problem, including adaptive task assignment problem. For instance, in Mofrad et al. (2019), some authors of this paper discuss that the point location can represent the difficulty level of a task that a participant can handle, and tries to find that point as fast and accurate as possible. The participant performance in Mofrad et al. (2019) is modeled using a stair function with two levels: a high performance for difficulties under the optimal *manageable* difficulty level and a low performance for difficulties just above the same level, i.e., the *manageable* optimal difficulty level. However, if we rather use a more realistic performance function according to which the performance is continuous and monotonically decreases as a function of the difficulty level, the approach proposed in Mofrad et al. (2019) will basically converge to difficulty level for which the participant performance is at 50% under some mild conditions. In other words the model finds a manageable difficulty level and can be used in titrated delayed MTS, SRT and online environments. Such remark motivated the current study in which we resort to the latter realistic performance model, for efficiently finding a higher rates of performance that are motivating enough for the learner, and provides a balance between challenge and skills, usually above 50% such as 70%. In comparison with Mofrad et al. (2019), where the adjustment technique is symmetric, in the current work the effect of correct and incorrect responses are not the same, i.e. the adjustment is asymmetric.

## Modeling task selection as balanced game using balanced difficulty task finder

In this section, we present BDTF as the main contribution in this article which is a theory that aspires to learn the appropriate difficulty of a task rather than exploring different types of tasks as in the case of work in Andersen et al. (2016). Although both approaches can be combined, we clearly distinguish between them as the second case can be seen as a novel theory for determining the adequate difficulty level of an assignment for the purpose of keeping the learning activity *motivating* and not *exploring* (as in Andersen et al. (2016), which is more concerned about exploring the different tasks in a similar manner to bandit problem).

Difficulty is a subjective concept, or more precisely, it is more *individual* and personal (see, e.g. Gilbert et al. 2012). We argue that difficulty should be tailored to the ability of the student. In fact, as in video games, or chess, the player is motivated by an appropriate level of challenge or equivalently difficulty. For example, the purpose of Xbox TrueSkill system (Herbrich et al. 2006) is to match players that have similar capabilities so that the outcome of the game is unpredictable (optimally equi-chance of winning and losing). Elo tries to find a global ranking among players and TrueSkill is similar to the Elo rating system for matching chess players. We advocate that, in a similar manner to TrueSkill and Elo, a student needs to find an enough challenging assignment that matches his capabilities.

After a brief introduction on psychological concept of flow experience (“The concept of flow” section), reviewing related works on games (“Related work on games” section), and related works addressing neural basis of adaptive task difficulty and the state of flow (“Neural basis of adaptive learning and state of flow experience” section), we provide a sound mathematical formulation (“Formulating learning as a balanced difficulty game” section) that emanates from the field of stochastic approximation (Kushner and Yin 2003).

### The concept of flow

The history of optimal human functioning in humanistic and health psychology can be tracked back to the work of Maslow (1959) who refereed to these moments of self-actualization *peak experiences*. These experiences are described as instances of happiness, fulfillment, and achievement with a feeling of awareness to one’s human potential. Csikzentmihalyi (1996) has described such an experience as a state of flow since it is characterized by *“an almost automatic, effortless, yet highly focused state of consciousness”* (p. 110).

Any mental or physical activity, according to Csikzentmihalyi (1996), can generate flow if: it is a challenging enough task that requires intense concentration and commitment, involves clear goals, provides immediate feedback, and is perfectly balanced to the skill level of the person.

**Fig. 1:**
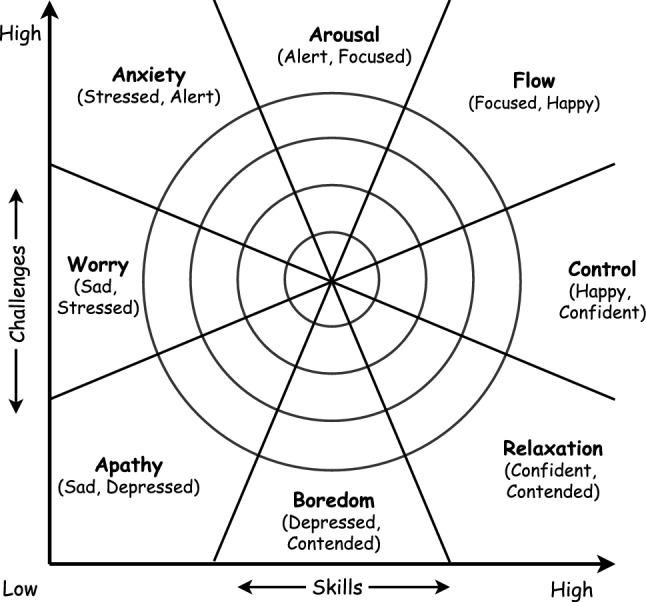
Model of the flow state adapted from Csikszentmihalyi (2020). Perceived challenges and skills must be above the person average level in order to experience a state of a flow. The apathy is the case when both are below the average and the experience intensity is increased by distancing from average, shown by rings

Delle Fave and Massimini (1988) discuss that balancing challenges and skills is not enough for optimizing the quality of experience and the notion of *skill stretching* inherent in the flow concept. They redefined flow as the balance of challenges and skills at the time both are above average levels for the person. Moreover, the quality of experience intensifies in a channel by moving away from a person’s average levels in the challenge/skills space. Figure 1 depicts a classification of experiences based on the level of challenge and skill in eight categories. The rings depict increasing intensity of experience in each channel or quadrant (see Nakamura and Csikszentmihalyi 2014, for a detailed overview of the concept of flow).

### Related work on games

A representative study that sheds light on the relationship between three inter-related concepts: difficulty, motivation and learning is presented by Chen (2007) that introduces the flow Channel to the filed of games. According to Schell (2014) and Chen (2007), when the difficulty exceeds the learner’s skill, the learner experience a feeling of anxiety at the thought of his learning skills are insufficient, and as a result gets demotivated. Consequently, the learner tends to abandon the activity after short time. On the other hand, boredom takes place in the other extreme case where the student level is much higher than the assignment’s difficulty. In this sense, the student perceives the assignment as a waste of time. The ideal case according to Schell (2014) and Chen (2007) takes place when the aptitude of the learner and the difficulty level are in *state of balance*. In this case, similar to the psychological definition of flow, the learner is said to achieve a state of flow. Chen (2007) defines the flow as: “the feeling of complete and energized focus in an activity, with a high level of enjoyment and fulfillment”.

As reported by Gallego-Durán et al. (2016), the notion of difficulty in games does not seem to have attracted much attention in the field of education in general. In this perspective, the proposed BDTF tries to bridge the gap between two seemingly disjoint fields of research, namely, ITSs and game ranking/matching systems.

The most pertinent work to our approach emanates from the realm of computer games and chess where it was remarked that when the level of the game is either too difficult or too easy, the players abandon playing (Chen 2007; Schell 2014). Extensive literature has been centred on the design of adaptive method to adjust the difficulty of the game so that to match the level of the players, but in the interest of brevity, we skip them (see, e.g. Hunicke 2005).

### Neural basis of adaptive learning and state of flow experience

There are many studies on the neural basis of state of flow that we briefly review some of them. Due to the complexity of concept of flow, it must be measured through its components. Dietrich (2004) analyses the flexibility/efficiency trade-off in the flow state and concludes that a prerequisite to the experience of flow period is “a state of transient hypofrontality that enables the temporary suppression of the analytical and meta-conscious capacities of the explicit system”. Klasen et al. (2012) use brain imaging to study neural basis of flow and showed an influence of flow on midbrain reward structures as well as complex network of sensorimotor, cognitive and emotional brain circuits. Some of the components of flow that identified in this study are focus, direct feedback, balance between skill and difficulty, clear goals and having control over the activity. Flow association with prefrontal functions such as emotion and reward processing was suggested by Yoshida et al. (2014) where brain activity in the prefrontal cortex during a flow state is examined using functional near-infrared spectroscopy (fNIRS). Cheron (2016) addresses some possible ways to measure the psychological flow from a neuroscience perspective. The neuroscience studies on games are not limited to the flow state, but we leave it since it is out of the scope of this article (see Palaus et al. 2017, for a systematic review on neural basis of video gaming).

To achieve and keep the state of flow, we use adaptive task difficulty methods. The neural basis of adaptive task difficulty has been studied by researches of the field (see, e.g. Flegal et al. 2019). An important issue is to see if the cognitive training effect could transfer to untrained tasks and neural plasticity. Kalbfleisch et al. (2007) study the influences of task difficulty and response correctness during fluid reasoning on neural systems using functional magnetic resonance imaging (fMRI). Von Bastian and Eschen (2016) compared conditions in which the difficulty of working memory training tasks was adaptive, self-selected, or randomly varied, in a behavioral study. The reported results indicate that all three procedures produced equivalent improvement on trained tasks, in comparison with an active control group. However, no significant difference between the training groups and the active control group, was reported for the transfer effects on untrained working memory tasks and far transfer (reasoning) tasks. So the transfer effects could not link to adaptivity or variability of task difficulty. McKendrick et al. (2014) examined mechanisms of training-induced plasticity by comparing a group that received adaptive working memory training with an active control group where task difficulty was matched to the performance of participants in the adaptive group, i.e. training was variable but not individually adaptive. The method was continuous monitoring of working memory training with near infrared spectroscopy (NIRS) during a dual verbal–spatial working memory task. The results suggested refuting the hypothesis that the effectiveness of adaptive task difficulty and variable task difficulty are alike. Flegal et al. (2019) study the effect of adaptive task difficulty on transfer of training and neural plasticity by measuring behavioral and neural plasticity in fMRI sessions before and after 10 sessions of *working memory updating (WMU)* training. The tasks difficulty was either fixed or adaptively increased in response to performance. The results show the transfer to an untrained episodic memory task activation decreases in striatum and hippocampus on a trained WMU task in adaptive training. Flegal et al. (2019) support the use of adaptive training as the best practice and suggest that cognitive training programs need to incorporate adaptive task difficulty to extend the transfer of training gains and optimize the efficiency of task-related brain activity (see also Gaume et al. 2019; Mora-Sánchez et al. 2020, for brain-computer interfaces which are able to monitor the working memory load and cognitive load in real-time based on biomarkers derived from EEG).

### Formulating learning as a balanced difficulty game

Without loss of generality, we suppose that the difficulty of any given task can be characterized by a real number from [0, 1], where 0 denotes the lowest possible difficulty and 1 denotes the highest possible difficulty.

The main intuition behind BDTF is the fact that the chance of a student for succeeding in a given task decreases monotonically as the difficulty level increases. We suppose that a student possesses a characterizing skill-curve that describes the relationship between the difficulty of the task and the student chance for succeeding in solving the task. We assume that the tasks are ranked on scale from 0 to 1 by an expert such as teacher where 0 denotes the lowest level of difficulty and 1 denotes the highest level of difficulty.

We suppose that $s^{\ast}$ is the optimal success probability that we want a learner (student) to experience. It is up to the designer of the intelligent tutoring system to fix the desired target chance of the succeeding in a task for a student. Thus, our approach will try to adjust the difficulty of the given tasks in an online manner that drives the system towards a state of flow (Chen 2007). Inspired by Elo system, one can choose $s^{\ast}=0.5$ which basically means that the designer desires that the student finds the tasks challenging enough by fixing the target success probability to 50%.

Please note that this reflects the most uncertain case since the outcome of the task in terms of success or failure is unpredictable. However, deciding on $s^{\ast}$ value requires more in depth study that takes into account many factors including psychological factors. In this paper, and in all the experiments presented in the rest of the article, we will fix $s^{\ast}=0.7$ which basically reflects the fact that we desire the student to succeed most of the time in solving the given task while failing just 30% of the time.

In addition, we suppose that we are operating in a discrete time space and *t* referring to the current time instant. The difficulty of the next assignment at time $t+1$ depends on the difficulty of the solved assignment at time *t* as well as the previous achievement (success or failure).

1 $$\displaystyle d(t+1) = \left\{ \begin{array}{ll} \min (1,~ d(t)+\lambda (1-s^{\ast})){:} &{} \quad {\text {if }} x(t)=1 \\ \max (0,~ d(t)-\lambda s^{\ast}){:} &{} \quad {\text {if }} x(t)=0 \end{array} \right.$$

where *d*(*t*) denotes the difficulty of the task at time *t*,^7^$\lambda$ is an update parameter that is in the interval ]0, 1[, and *x*(*t*) denotes the binary variable that records the result of solving the task given at time instant *t*. $x(t)=0$ in case of failure and $x(t)=1$ in case of success.

Equation (1) describes a recursive update of the difficulty of the tasks depending on the performance of the student, *x*(*t*). According to Eq. (1), the difficulty gets increased upon success and decreased upon failure in an asymmetric manner. We suppose that at time $t=0$, the BDTF starts by suggesting a task with difficulty $d(0) = 0.5$, i.e, we start with tasks with *medium level*. We suppose that for student *i*, there is a function $S_i(d)$ that describes the success probability given the difficulty of the task. Whenever there is no ambiguity, we drop the index *i*. As explained previously, the latter function is monotonically decreasing. Please note that $x(t)=1$ with probability *S*(*d*(*t*)) and $x(t)=0$ with probability $1-S(d(t))$. We will later provide theoretical results that demonstrate that if there exists a point $d^{\ast}$ such that $S(d^{\ast})=s^{\ast}$ then the update equation converges to it. Since *d* is defined over [0, 1] and *S*(*d*) is decreasing over [0, 1] and admits values in [0, 1], then for any function $S_i$ such point $d^{\ast}$ is unique (if it exists). A simple and sufficient condition for the existence as well as uniqueness of $d^{\ast}$ is that $S_i(0)=1$ and $S_i(1)=0$. This has an intuitive interpretation: the success probability for the $\min$ difficulty is one and for the $\max$ difficulty is zero. However, in general, *S*(0) might be different from one and *S*(1) might be different from zero. The following theorem catalogues the convergence of our scheme for an arbitrary monotonically decreasing function *S* such that *S* is mapping from [0, 1] to [0, 1].^8^

It is noteworthy that the proof of the coming theorem is based on the results of the stochastic approximation theory (Kushner and Yin 2003). The informed reader would observe that our algorithm is very similar to the seminal algorithm of Robbins and Monro (1951) who pioneered the field of stochastic approximation. The main differences are the following:

- They use a time dependent update parameter $\lambda$.
- In Robbins and Monro (1951), the response function is increasing, while in our case it is decreasing.

Those differences can be tackled easily in the proof as within the field of stochastic approximation, there are two types of algorithms: algorithms with fixed step size and algorithms with time varying step size, usually decreasing. We are working in this paper with a fixed step size algorithm. The second difference concerns the response function. The monotonicity of the function gives uniqueness of the equilibrium. If our function was increasing, we would simply change $\lambda$ by $-\lambda$. This form of update is similar to gradient descent where the direction of movement is determined according to whether we are facing a minimization or maximization problem.

#### **Theorem 1**

*The stochastic process* *d*(*t*) *converges to one of the three following cases as the learning parameter* $\lambda$*tends to zero:*

- *Case 1* *If* $\min S(d) \le s^{\ast} \le \max S(d)$, *then* $\lim _{t \rightarrow \infty } \lim _{\lambda \rightarrow 0} d(t) = S^{-1}(s^{\ast})=d^{\ast}$.
- *Case 2* *If* $\max S(d) <s^{\ast}$, *then* $\lim _{t \rightarrow \infty } \lim _{\lambda \rightarrow 0} d(t) = 0$.
- *Case 3* *If* $\min S(d) >s^{\ast}$, *then* $\lim _{t \rightarrow \infty } \lim _{\lambda \rightarrow 0} d(t) = 1$.

#### *Proof*

Similar to Altman et al. (2009), we can re-write the update equations as per:

2 $$\begin{aligned} d(t+1)= \Pi _H(d(t)+\lambda (x(t)-s^{\ast})) \end{aligned}$$

where $\Pi _H$ denote the following projection

$$\Pi _H(d)= {\left\{ \begin{array}{ll} d, &{}\quad {\text {if}} \,\,1<d<0,\\ 1, &{}\quad {\text {if}}\,\, d\ge 1,\\ 0, &{}\quad {\text {if}}\,\, d\le 0. \end{array}\right. }$$

The usage of projection is common with the field of stochastic approximation to force the iteration to stay with a bounded set $H=[0,1]$, and they are projected back to the set whenever they go outside it. Without loss of generality, the boundary set we are using here, consisting of zero and one, is a well-behaved one as described by Borkar (2009, Chapter 5.4). We can show that process converges to some limit set of the following Ordinary Differential Equation (ODE):

3 $$\begin{aligned} {\dot{d}}= E[x(t)|d] -s^{\ast}. \end{aligned}$$

We know that $E[x(t)|d]=S(d)$, therefore the ODE is

4 $$\begin{aligned} {\dot{d}}= S(d) -s^{\ast}. \end{aligned}$$

The decreasing nature of *S*(*d*) provides the uniqueness of the fixed point $s^{\ast}$ whenever $\min S(d) \le s^{\ast} \le \max S(d)$. Whenever $s^{\ast}$ lies outside $H=[0,1]$, we will converge towards the boundary point, zero and one, according to whether $\max S(d) <s^{\ast}$ or $\min S(d) >s^{\ast}$ respectively.□

## Experimental results

In this section, we provide some experimental results which confirm the theoretical results presented in Theorem 1.

In order to describe the relationship between difficulty and success, we define $S(d)=a - b/(1+\exp (-\,20*(d-0.5)))$, where $0 <b \le a \le 1$, ensuring that *S* is decreasing. In the reported results for three cases of the theorem, $\lambda = 0.01$, and the target success probability is $s^{\ast}=0.7$. Please note that the aim of the section is to rather confirm the theoretical properties of our scheme so any decreasing function suffices.

**Fig. 2:**
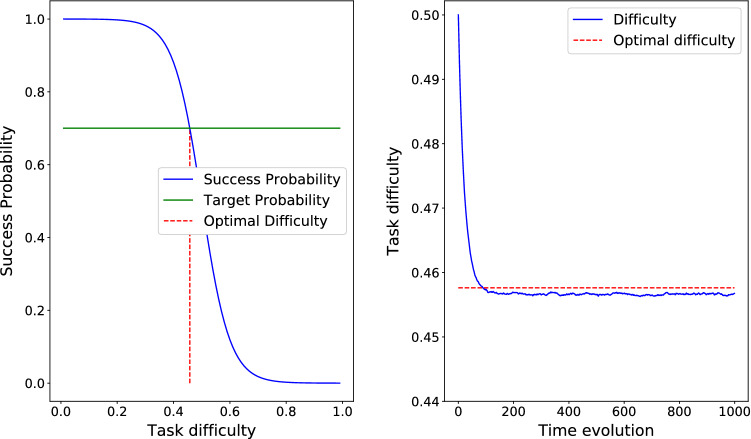
Case 1 in the theorem. $S(d) = 1 - 1/(1+\exp (-\,20*(d-0.5)))$ so *d*(*t*) converges to $d^{\ast} = S^{-1}(s^{\ast})=0.458$

Figure 2 depicts the time evolution of *d* and the corresponding success probability *S*(*d*) where $S(d)=1 - 1/(1+\exp (-\,20*(d-0.5)))$ for an update parameter $\lambda =0.01$. Please note that since $\min S(d)=0 \le s^{\ast}=0.7 \le \max S(d)=1$, then according to Theorem 1, *d*(*t*) converges to $d^{\ast}=S^{-1}(s^{\ast})=0.458$.

**Fig. 3:**
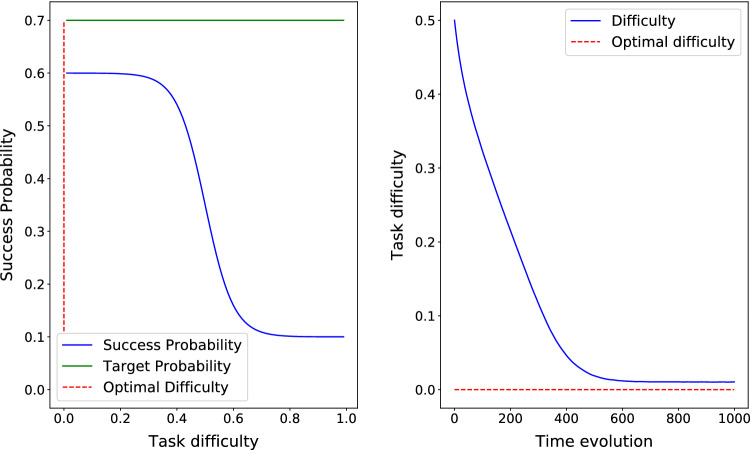
Case 2 in the theorem. $S(d)= 0.6 - 0.5/(1+\exp (-\,20*(d-0.5)))$ so *d*(*t*) converges to $d^{\ast}=0$

Figure 3 depicts the time evolution of *d* and the corresponding success probability *S*(*d*) where $S(d)=0.6 - 0.5/(1+\exp (-\,20*(d-0.5)))$ for an update parameter $\lambda =0.01$. Please note that since $\max S(d)=0.6 < s^{\ast}=0.7$, then *d*(*t*) converges to $d^{\ast}=0$.

**Fig. 4:**
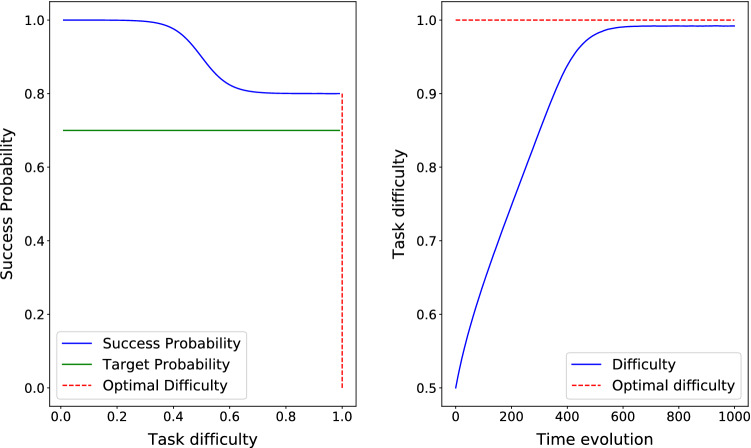
Case 3 in the theorem. $S(d)= 1 - 0.2/(1+\exp (-\,20*(d-0.5)))$ so *d*(*t*) converges to $d^{\ast}=1$

Finally, Fig. 4 depicts the time evolution of *d* and the corresponding success probability *S*(*d*) where $S(d)=1 - 0.2/(1+\exp (-\,20*(d-0.5)))$ for an update parameter $\lambda =0.01$. Since $\min S(d)=0.8 > s^{\ast}=0.7$, then *d*(*t*) converges to $d^{\ast}=1$.

**Fig. 5:**
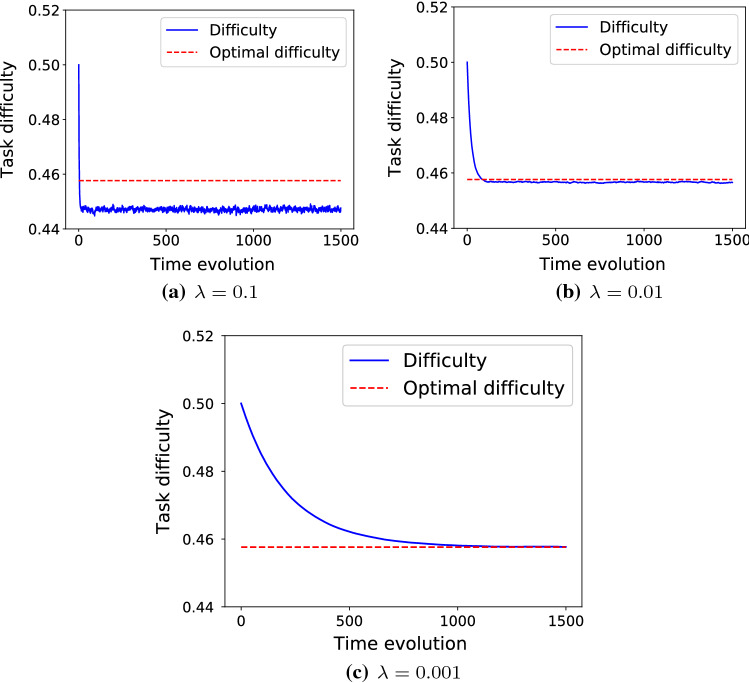
The success probability function based on difficulty is $S(d) = 1 - 1/(1+\exp (-\,20*(d-0.5)))$, which is depicted in Fig. 2. The optimal task difficulty for success probability $s^{\ast}=0.7$ is $d^{\ast} = 0.458$ and shown by dashed red line

Please note that the convergence time is a function of both starting point distance to optimal difficulty and value of $\lambda$. In Fig. 2, the optimal difficulty is $d^{\ast}=0.458$ which means it is about 0.14 far from the starting point. After around 100 iterations, the optimal difficulty is reached. In Figs. 3 and 4 the optimal difficulty is about 0.5 far from the starting point, and in both cases after about 600 steps, the optimal difficulty is reached. In all the three cases, $\lambda = 0.01$. To study the role of $\lambda$ in the convergence time, we fix the success probability function to $S(d) = 1 - 1/(1+\exp (-\,20*(d-0.5)))$, which is depicted in Fig. 2 and test it for three different values of $\lambda = 0.1$, $\lambda = 0.01$, and $\lambda = 0.001$. As we see in Fig. 5, smaller values of $\lambda$ result into a slower, but smoother convergence. In Fig. 5a, with $\lambda =0.1$, the convergence is just about 10 steps, in Fig. 5b, with $\lambda =0.01$, the convergence happens after about 100 steps, and finally in Fig. 5c, with $\lambda =0.001$, the convergence happens after about 1000 steps. Hence, the value of $\lambda$ can be chosen in a way to find a compromise between convergence speed and convergence accuracy.

**Fig. 6:**
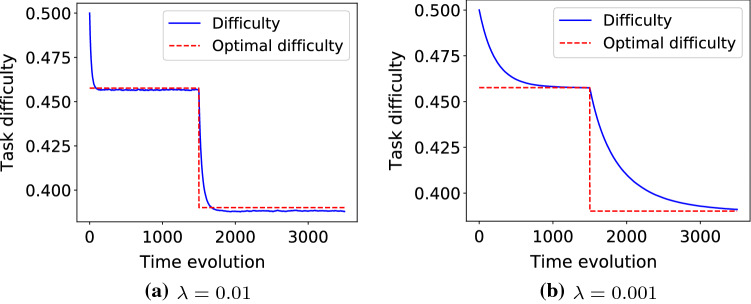
The success probability function based on difficulty is $S(d) = 1 - 1/(1+\exp (-\,20*(d-0.5)))$, which is depicted in Fig. 2. The optimal task difficulty for success probability $s^{\ast}=0.7$ is $d^{\ast} = 0.458$ in the first 1500 time instances, then the target success probability changes to $s^{\ast}=0.9$ which means the optimal task difficulty is $d^{\ast} = 0.39$. The optimal task difficulty is shown by dashed red line

The aim of the last experiment is to demonstrate the ability to track the changes in optimal difficulty. This is analogous to the cases where instructor or teacher decides to give easier or harder tasks based on the feedback from learner. In Fig. 6 the optimal success probability is set to $s^{\ast}=0.7$ at the beginning where the learner achieves this success rate when the optimal difficulty is $d^{\ast} = 0.458$. Then at time instance $t=1500$, the teacher see that this is still challenging for the student and decided to provide student with tasks that 90% of the time handled by student. Figure 6a shows the case that $\lambda = 0.01$ and therefore the change rate it higher. Figure 6b is when changes are slower, $\lambda = 0.001$. The optimal difficulty for $s^{\ast} = 0.9$ equals $d^{\ast} = 0.39$.

## Conclusions and future work

In this paper, we tackled the problem of personalized task assignment in online learning environment as well as training methods for retaining information. We present the BDTF which is a formal theory by which an ITS can fine tune the difficulty of a task to a level that matches the student level. The underlying assumption of the BDTF is that the ITSs can fine tune the difficulty of the task to a continuous level. The BDTF application to the learning methods that focus on memory and retaining information such as adjusting delayed MTS and spaced retrieval training methods is discussed. These methods are looking for the best delay time between two consecutive tasks and can be used for memory training.

The BDTF approach deals only with binary feedback. It is possible to extend our work so that to accommodate non-binary feedback in the form of a continuous or discrete score reflecting the achievement of the student in solving a given task. Furthermore, as a future work, we intend to explore the effect of learning on the progress of the student. Intuitively, the success probability *S*(*d*) shall also be frequency dependent, i.e, the more assignments the student tries, the higher the chance of success in future tasks. This is also described as the learning effect that results from repetitive trials. The latter effect can be easily accommodated in our model by rendering *S*(*d*) a function of the number of trials, meaning the dynamics of *S*(*d*) shall include a frequency dependent term. An interesting avenue for research is the possibility of introducing the recency and spacing in time between the different student trials as an extra parameter in *S*(*d*). BDTF approach could be extended to the tutorial-like systems similar to the LA applications for a generalized concept of ITS proposed by Hashem (2007). Since we are using LA, we can integrate the idea of having an stochastic teacher (Hashem and Oommen 2007), modeling a classroom of students where artificial students can interact and learn from each other as well as the teacher (see Oommen and Hashem 2009a, for such a model), and propose an adaptive learning model of teacher and how teaching abilities of a teacher can be improved during the process (inspired by Oommen and Hashem (2013)).

## Footnotes

1 www.khanacademy.com.

2 www.udemy.com.

3 https://ocw.mit.edu.

4 www.bakpax.com.

5 www.codecademy.com.

6 Matching-to-sample procedures, have been frequently used to study complex human behavior (see for instance Cumming and Berryman 1965; Sidman 1994). Arntzen (2012) provides an overview of MTS experiments and several variables that can be manipulated when designing an experiment through MTS procedures. In adjusting delayed MTS, the length of the delay changes as a function of the participants’ responses, which makes it similar to the adaptive task assignment problem.

7 When relation to time is not important, we simply use *d* to refer to difficulty.

8 The function *S*(.) has values within [0, 1] since it denotes the probability of success.
